# A Novel Method for Changing the Dynamics of Slender Elements Using Sponge Particles Structures

**DOI:** 10.3390/ma13214874

**Published:** 2020-10-30

**Authors:** Mateusz Żurawski, Bogumił Chiliński, Robert Zalewski

**Affiliations:** Institute of Machine Design Fundamentals, Warsaw University of Technology, 02-524 Warsaw, Poland; Bogumil.Chilinski@pw.edu.pl (B.C.); Robert.Zalewski@pw.edu.pl (R.Z.)

**Keywords:** mass redistribution, sponge particles structure, semi-passive damping, experimental research

## Abstract

The paper concerns problems related to controlling the dynamic properties of beam-like elements. The parameters of the investigated system can be changed by external factors, resulting in partial changes in the system mass redistribution. It is assumed that it is possible to control the system dynamics by shaping the object frequency structure. The paper introduces the mathematical model of the investigated cantilever beam filled with a Sponge Particle Structure. The continuous model has been simplified to a discrete multi-degree of freedom system. The influence of the system parameters on its behavior is discussed in details. The possible applications of the presented concept are proposed. The spectral vibration analyses were carried out. Theoretical considerations enabled the use of the preliminary semi-active method for controlling the vibration frequencies through a mass redistribution. Experimental studies were carried out to verify the proposed mathematical model.

## 1. Introduction

Vibrations are important phenomenon which cause many undesired effects. There are a lot of problems related to operating various types of devices in a resonant range [1]. Harmful and dangerous vibrations are generally attenuated using passive [2,3] and active methods [4,5]. Nowadays, semi-active vibrations attenuation techniques are also getting popular [6]. The most sophisticated, complex and, in consequence, expensive technique is an active method [7]. Unfortunately, it requires an additional power supply, sophisticated measuring system and very often does not fulfill costs and environment limitations. The obvious alternative for a previously mentioned method is a passive strategy. Various approaches to passive vibration attenuation exist. The most popular is frictional, viscous and impact damping [8]. The main disadvantages of the viscous (viscoelastic) and frictional damping are nonlinear characteristics or strong degradation effects [9]. Concluding, the most interesting and universal seem to be impact dampers. In this group of devices, Particle Impact Dampers (PIDs) play the most important role [10,11,12]. Problems related to the damping of vibrations are popular in publications in the field of civil and mechanical engineering. Therefore, in this paper the authors will limit themselves to a brief description of vibration attenuation methods using granular materials [13]. A granular damping technique shows some similarities to classical impact damping. When the impact damping involves the movement of a single additional mass in the vibrated system, the particles damping refers to many auxiliary masses with relatively small sizes, moving in a specially-shaped cavity. The principle of the Particle Impact Dampers is the dissipation of energy of the vibrating element through inelastic collisions occurring between individual grains and between grains, and the container in which they are located [11]. Another phenomenon existing in described energy attenuators is friction occurring as an ”intergranular” force. The main advantages of granular dampers are low sensitivity to temperature changes [14], long life, reliability in a wide range of frequencies, reduction in the global mass of the attenuated system (the grains often have lower mass than the mass of the initial structure they replace), and low sensitivity for operation in a harsh environment [15,16]. The main disadvantage of PIDs is the passive means of vibration damping [17]. The adjustment of the damping properties is acquired by the selection of a proper size, material and filling ratio of particles. Detailed analyses of PIDs behavior is mainly based on empirical tests [18]. The results of experiments are the base for numerical simulations carried out mainly using the Discrete Element Method (DEM) or Finite Element Method (FEM) [19]. Analytical approaches seem to be less popular. Although the grain interaction mechanics are quite simple, such an approach demands a high computing power. Conducting numerical tests for thousands of grains is a challenge even for modern and effective computers. Over the last three decades, many papers have revealed the results of research carried out in the field of granular energy attenuators. Interesting research results were discussed in [20], where the system consisting of 10,000 particles was taken into consideration. Loose grains were moving in the cavity accordingly to various external loading forces. The damping efficiency of such a system was investigated in detail. In [21], a mathematical model was developed that allows one to predict the behavior of granular damper. The applied mathematical formulation describes the grains dynamics and takes into account the viscous properties of the grain materials. Additionally, a friction phenomenon occurring in the contact zones was involved. An interesting application of the granular damper for the vibrations attenuation of a cantilever beam is presented in [22]. The authors revealed preliminary laboratory tests results. Based on experimental data, it can be stated that capturing the real behavior of the highly nonlinear granular system is challenging. The verification of simulation results carried out, based on the proposed mathematical model with real empirical data, seems to be promising. A PID design methodology has been discussed in [23,24]. The authors proposition limits and many typical problems encountered in such devices. It is underlined that granular dampers have strongly nonlinear damping characteristics. Interesting standards for designing particles dampers are presented. The guidelines simplifying the selection of the proper granular damper for the vibrated systems are presented and verified experimentally.

Not only are loose granular materials applied in damping strategies. An interesting proposition of the semi-active damping of vibrations strategy, including so called Vacuum Packed Particles, are presented in previous works of the authors. In [25,26] it is confirmed that the underpressure value can be an efficient parameter of controlling the dissipative properties of a cantilever beam subjected to various types of excitations.

In this paper, the original method of slender elements (shafts, beams or rods) natural frequency shaping with an application of the local mass redistribution is presented. The global concept is to change the vibrating object mass distribution and to affect its dynamics. Such a solution seems to be possible thanks to the application of innovative granular structures. The idea of the proposed method is to divide the beam into several hermetic sections. Each segment is filled with a structure that enables its density to be changed. In this paper, the authors assumed that Sponge Particles Structures (SPS) can be a filling material. Such a structure consists of loose grains embedded in soft sponge layers that exhibit various mechanical properties according to changes in the external pressure. The proposed shaping of the dynamic features strategy is novel and promising. In this paper, the authors assume that the external pressure provided to the structure by the system of balloons can be an efficient parameter to reorganize the grains inside each section, causing the changes in the mass redistribution of the investigated element.

**Figure 1:**
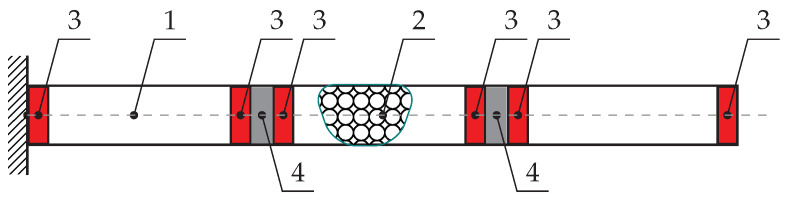
The idea of the investigated system (1—shell type beam structure; 2—Sponge Particles Structure (SPS); 3—balloons; 4—fixed diaphragms).

The concept introduced in the paper involves a rectangular shell-like cantilever beam. Inside the object, two fixed partitions are mounted. The system additionally consists of a Sponge Particles Structure and six balloons. The discussed idea is depicted in Figure 1.

Inflating balloons (3) results in compressing the SPS (2) placed between them. Changing the internal pressure in the balloons enables the granular mass embedded in a sponge matrix to be compressed and redistributed. This phenomenon is a convenient way to change the dynamical properties of the investigated system. For the purpose of mass redistribution, not only SPS can be involved. Additionally, other unconventional structures, such as magnetorheological fluids or elastomers can be useful [27,28]. The volume of the internal sections can also be changed mechanically (by screws), hydraulically, or by the external magnetic field. The paper presents a novel adaptive–passive (semi-active) method for damping of vibrations. The main objective is to confirm the effectiveness of the mass redistribution method for a controlling process of the beam structures dynamics. The proposed methodology can be applied in various practical engineering applications. It is worth mentioning that such an adaptive damping strategy can be potentially used in structures, such as in the wings of aircrafts, slender skyscrapers, long bridge spans, or supports of wind turbines.

## 2. Preliminary Modeling and Analysis of Mass Redistribution on the System Dynamics

Based on the mathematical analysis of the continuous systems, it is possible to show that the models with infinite degrees of freedom can be reduced to the finite number of nodes. Such a simplification allows for easy and quick dynamics analyses. However, an inappropriate discretization of the problem will lead to incorrect results. Therefore, in the preliminary stage of the presented research, the impact of Degree of Freedom (DOF) number on eigenvalues and eigenmodes was numerically tested. In order to perform eigenfrequencies validation, the beam was divided into arbitrary given number of segments. For each segment, equivalent mass and stiffness were obtained. Based on determined data, inertia and stiffness matrices were computed. It enables one to find eigenfrequencies of the given system and to investigate the impact of node numbers on the computational results. Based on the aforementioned methodology, the natural vibration frequency of the fixed beam reduced to 3, 5, 7, 9 and 10 nodes was calculated.

Governing equations were rearranged to the eigenvalue problem form, in order to determine natural frequencies of the system. The solution was predicted in the form of Formula (1).

(1) X(t)=A·sin(ωt+φ)

After necessary substitutions and rearrangements, the following form of the eigenvalue equation can be obtained:

(2) $$\left(M^{-1}\;\cdot \;K\right)A=\omega^{2}\;\cdot \;A$$

The roots of the Equation (2) yield the natural system frequencies. The results of computations are shown in Table 1.

Analyzing the results, it can be concluded that the vibration frequencies of a system with 3 and 10 degrees of freedom differ by less than 15%. The authors assumed that such values in the context of further calculations are acceptable at the initial stage of investigations. To ensure the possibility of using a simplified beam model with 3-DOF, additional mathematical analyses were carried out. The frequency of natural vibrations for the system was determined and compared in which 60% of the entire system mass was moved to the end of the beam. The results are shown in Table 2.

Analyzing the data presented in Table 2 the authors assumed that for the preliminary numerical tests simple 3-DOF beam model is sufficient. The difference between responses of 3-DOF and 10-DOF models differs by less than 15%.

Every single linear dynamic system can be described by a second order differential equations. In the case of a multi DOF system it is possible to perform a modal decomposition and transform the problem to a simpler form obtaining the system of uncoupled equations. Then every mode can be treated as a single degree of freedom. A governing equation is given by (3).

(3) $$\ddot{x}+\omega_{0}^{2}\;\cdot \;x=A\;\cdot \;sin\left(\Omega \;\cdot \;t\right)$$

where:

$\ddot{x}$—acceleration;
$\omega_{0}$—natural frequency;
*x*—displacement;
*A*—excitation amplitude;
Ω—excitation frequency.

Dynamics of the system with varying parameters is important from the practical point of view. In the initial stage of investigations, the following problem of step changing parameters was analyzed. The transient process is described by Heaviside’s step function, which provides appropriate differential properties (for the adopted definition of the distributional derivative). It represents a sudden change of the system parameters, what is the system’s worst case scenario. Although it is a simple model of such a phenomenon, it allows for an efficient analysis of the case under investigation (from the analytical and numerical point of view). The formula describes a mass change which is presented in (4).

(4) $$m\left(t\right)=m_{0}\;\cdot \;\left(1+H\left(t-t_{0}\right)\right)$$

where:

m(t)—varying mass;
$m_{0}$—initial mass;
$H(t-t_{0})$—Heaviside function;
$t_{0}$—redistribution process starting time.

For that case, the governing equation is as follows:

(5) $$\ddot{x}+\omega_{0}^{2}\;\cdot \;{\left(1+H\left(t-t_{0}\right)\right)}^{-1}\;\cdot \;x=A\;\cdot \;{\left(1+H\left(t-t_{0}\right)\right)}^{-1}\;\cdot \;\sin\left(\Omega \;\cdot \;t\right)$$

Equation (5) is an ordinary differential equation with discontinuous parameters. It is a distributional problem which can be solved using generalized functions. For the problem under consideration, the following particular solution was predicted:

(6) $$\begin{array}{c} x\left(t\right)=\left(A_{1}\cos\left(\Omega \;\cdot \;t\right)+B_{1}\sin\left(\Omega \;\cdot \;t\right)\right)\;\cdot \;H\left(t_{0}-t\right)+\\ +\left(A_{2}\cos\left(\Omega \;\cdot \;t\right)+B_{2}\sin\left(\Omega \;\cdot \;t\right)\right)\;\cdot \;H\left(t-t_{0}\right) \end{array}$$

Based on the predicted solution (6), the parameters $A_{1}$, $B_{1}$, $A_{2}$ and $B_{2}$ were found.

(7) $$\begin{array}{c} A_{1}=\frac{a_{0}}{\Omega^{2}-\omega_{0}^{2}}\\ B_{1}=0\\ A_{2}=\frac{a_{0}}{\Omega^{2}-0.5\cdot \omega_{0}^{2}}\cdot \tan^{-1}(\frac{\sin(t_{o})}{\cos(t_{o})})\\ B_{2}=\frac{a_{0}}{\Omega^{2}-0.5\cdot \omega_{0}^{2}}\cdot \cot^{-1}(\frac{\sin(t_{o})}{\cos(t_{o})}) \end{array}$$

It should be noted that the damping parameter does not appear in the general mathematical solution (Equation (5)) of the proposed phenomenon. The term “damping” is often used in many papers and handbooks to describe the process of the reduction in vibration amplitudes. The idea of the proposed method is exactly the same as a working principle of vibration absorbers. These devices are often called Tuned Mass Dampers. That is why the authors decided to use the term “damping” to call the phenomenon of resonant frequency shifting.

## 3. A Proposition of Modeling and Shaping of Beams Dynamics

In general, case beam-like elements should be treated as continuous systems. Unfortunately, such an approach is quite complicated from the computational point of view. There are closed solutions for continuous shaft dynamics, but only for simple cases. For this purpose, it is necessary to find an equivalent inertia and stiffness matrix to start with a preliminary analysis of the continuous system. In the case under consideration, fundamental laws of linear-elasticity—e.g., Castigliano theorem or Maxwell-Mohr method—were used. Using Equations (8) and (9) it is possible to find flexibility coefficients:

(8) $$\delta_{ij}=\int_{0}^{l}\frac{M_{bi}^{\prime }M_{bj}^{\prime }}{EJ}dl$$

where:

$\delta_{ij}$—flexibility matrix;
$M_{bi}^{\prime }$—unit bending moment;
$M_{bj}$—bending moment;
*E*—Young modulus;
*J*—moment of inertia.

(9) $$K=\delta^{-1}$$

where:

*K*—stiffness matrix.

The Inertia matrix *M* can be derived with the application of mass conservation law or energy conservation of entire system. In the general case, it is possible that the matrix M will be diagonal (10).

(10) $$M=\mathrm{diag}(m_{1},m_{2},\ldots ,m_{n})$$

where:

*M*—inertia matrix;
*n*—number of nodes;
$m_{i}$—nodal masses.

**Figure 2:**
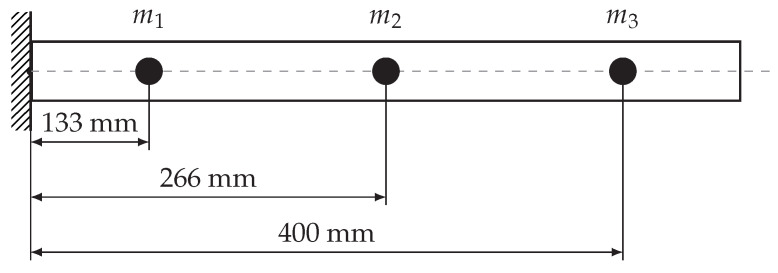
Mathematical model of the cantilever beam.

Natural frequencies can be found as eigenvalues of the matrix $M^{-1}K$. Four various inertia matrices were taken into account in the paper. The fixed beam with a circular cross-section was adopted as a model. The masses were concentrated at three different points as shown in Figure 2.

For further calculations, the following mass distribution values for individual nodes were adopted:

Basic mass distribution: $M_{base}=diag\left(2,2,2\right)$ kg;
First mass distribution: $M_{1}=diag\left(4,1,1\right)$ kg;
Second mass distribution: $M_{2}=diag\left(1,4,1\right)$ kg;
Third mass distribution: $M_{3}=diag\left(1,1,4\right)$ kg;
Youngs modulus: $E=2.1\times 10^{11}\;\mathrm{Pa}$;
Moment of inertia: $I=4.43\times 10^{-6}\;m4$;
Inner diameter of the beam: d=100 mm;
Outer diameter of the beam: D=116 mm.

To determine the influencing factors, the bending moment must be determined in advance. In the case under consideration (Figure 2), it can be described using the Heaviside function:

(11) $$\begin{array}{c} M_{b}=\left(x-L\right)\;\cdot \;H\left(x-L\right) \end{array}$$

The governing Equations (12)–(14) were determined based on obtained forms of the inertia and the stiffness matrices. The Lagrangian mechanics were utilized for this reason. The carried out computations revealed the influence of the mass changes. It has to be noticed that there is the impulse force, which comes from a sudden change in the mass during the redistribution process.

(12) $$\begin{array}{c} m_{1}{\ddot{x}}_{1}+{\dot{m}}_{1}{\dot{x}}_{1}+c_{1}{\dot{x}}_{1}+k_{11}x_{1}+\frac{k_{12}x_{2}}{2}+\frac{k_{13}x_{3}}{2}+\frac{k_{21}x_{2}}{2}+\frac{k_{31}x_{3}}{2}=f\left(t\right) \end{array}$$

(13) $$\begin{array}{c} m_{2}{\ddot{x}}_{2}+{\dot{m}}_{2}{\dot{x}}_{2}+c_{2}{\dot{x}}_{2}+\frac{k_{12}x_{1}}{2}+\frac{k_{21}x_{1}}{2}+k_{22}x_{2}+\frac{k_{23}x_{3}}{2}+\frac{k_{32}x_{3}}{2}=0 \end{array}$$

(14) $$\begin{array}{c} m_{3}{\ddot{x}}_{3}+{\dot{m}}_{3}{\dot{x}}_{3}+c_{3}{\dot{x}}_{3}+\frac{k_{13}x_{1}}{2}+\frac{k_{23}x_{2}}{2}+\frac{k_{31}x_{1}}{2}+\frac{k_{32}x_{2}}{2}+k_{33}x_{3}=0 \end{array}$$

**Figure 3:**
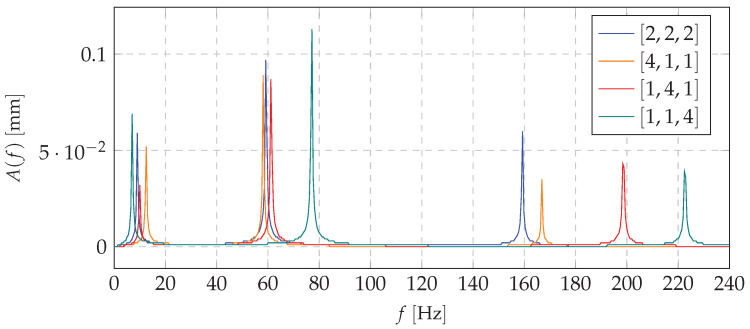
Resonance frequency for all cases of mass distribution.

Frequencies of natural vibrations in the investigated cantilever beam, including various mass redistribution cases, are presented in Figure 3.

Analysis of the results shows that the mass redistribution affects the dynamics of the entire system. However, it turns out that about 30% change in the inertia matrix shifts some frequencies by about 20%. In addition, it can be observed that the redistribution of mass to a particular location causes a significant change in the frequency of vibrations in other nodes.

## 4. Simulations

Obtained results confirmed the fact that the change in mass distribution will shift the resonant range. In the simulations, the harmonic force was introduced into the system. The investigated cantilever beam was subjected to various resonance loading frequencies. This allows us to observe the form of vibrations and to present a way for the mass distribution to influence the dynamics of the structure during operation. In Figure 3a, Frequency Response Functions (FRFs) for all cases under consideration are presented.

**Figure 4:**
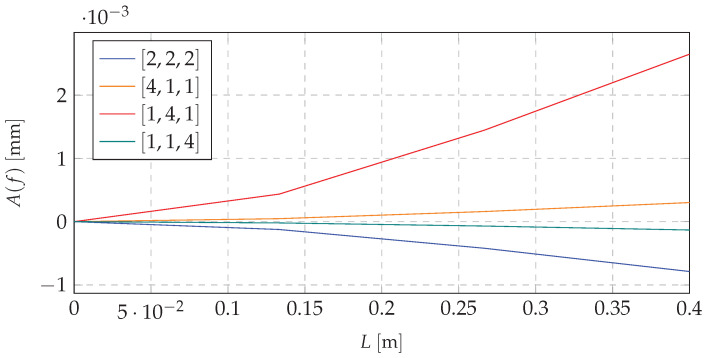
Resonance vibrations for 9.05 Hz and various mass redistribution cases.

**Figure 5:**
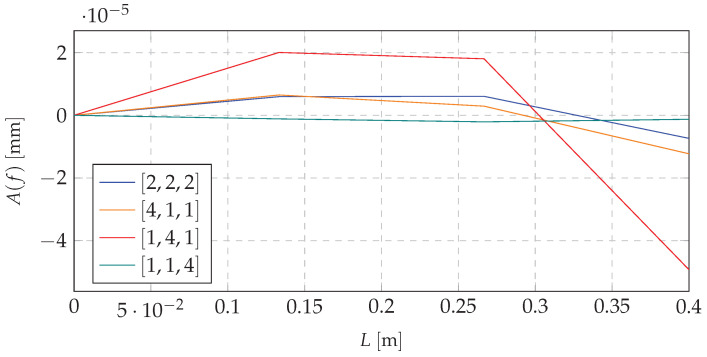
Resonance vibrations for 59.26 Hz and various mass redistribution cases.

Based on the mathematical model, and taking into account the excitation with the first natural frequency, the first forms of vibration were obtained. Shifting the mass to the proper node (choice of the node depending on the system dynamics and its initial mass distribution) decreases the amplitude of the dynamic response. It is possible to use that concept to switch the system states depending on the loading force pulsation. This approach ensures that the excitation forces will not cause a resonance or that it would appear in the short period of time. Figure 4 and Figure 5 present simulations for beam vibrations for all four redistribution cases, based on the resonance frequency of the [1,4,1] case of the first and second eigenvalue (9.05 and 59.26 Hz). Several integration procedures were applied, in order to carry out numerical simulations. The best results were obtained with Runge–Kutta fourth order algorithm.

**Figure 6:**
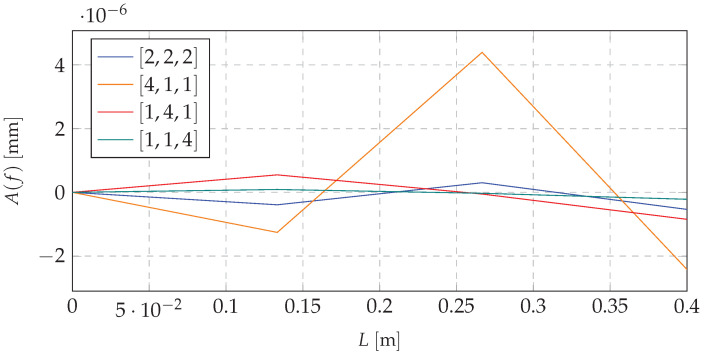
Resonance vibrations for 159.22 Hz and various mass redistribution cases.

Similar data for the third natural frequency are depicted in Figure 6.

**Figure 7:**
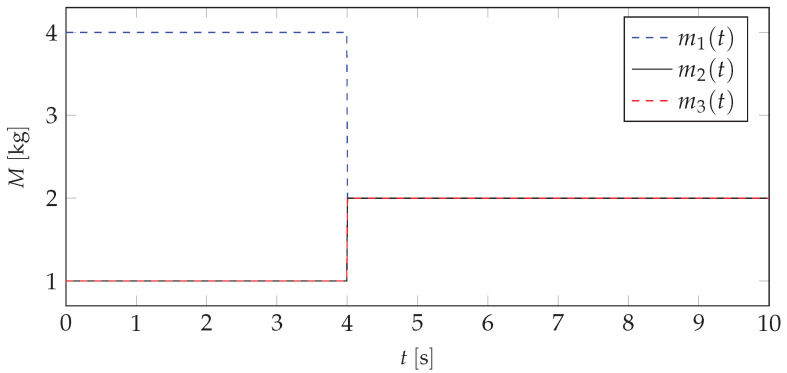
Functions of the masses changes for every node.

Analyzing the data depicted in Figure 4, Figure 5 and Figure 6, it can be stated that there is a possibility to avoid the resonant range by shifting the mass. It means that the mass redistribution process can be the basis for the efficient method of controlling the dynamics of the vibrating system. In the proposed method the mass transfer is carried out in a static way. Furthermore, simulations are based on the beam vibration analysis during the mass redistribution process. It means that the moving of the mass took a certain time. This causes the need for the investigation of the system behavior, depending on how the process of mass redistribution is carried out. Figure 7 presents time plot of masses changes at nodes during the system operation.

**Figure 8:**
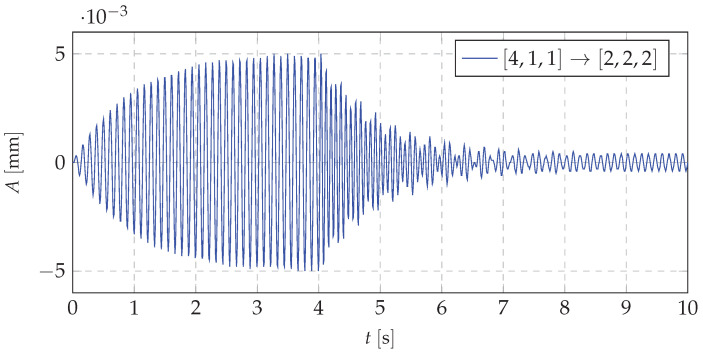
Deflection of the end of the beam during the process of mass redistribution in the neighborhood of the resonant vibration.

Typical results of vibration displacements of the beam end for a selected mass redistribution case ($M_{1}\to M_{base}$) are depicted in Figure 8.

**Figure 9:**
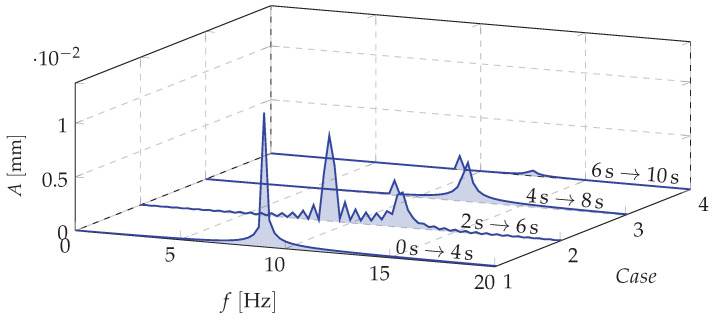
Short time spectrum of deflection vibrations—STFT.

The spectral analysis of vibrations displacements of the investigated cantilever beam during the dynamic mass redistribution process is depicted in Figure 9. Three types of vibrations can be distinguished: 1—vibrations of the beam with the mass being shifted to a specific point (node 1) (from 0 s to 4 s of simulation); 2—Beam vibrations during mass transfer (from 4 s to 8 s of simulation); 3—Beam vibrations with a homogeneous mass distribution (from 8 s to 10 s of simulation). Analyzing the data depicted in Figure 8a significant change in the displacements of the beam end can be observed. It could mean that the vibration frequencies during this process reach unexpected values. An amplitude spectrum of the beam vibrations (Figure 9) shows that the mass redistribution phenomenon causes ”blurring” of natural frequencies. It means that there is an intermediate process causing a smooth transition from the initial (in the resonance) to the final (beyond resonance) dynamic state. This is crucial because there is no risk of falling into other, unpredictable resonant ranges.

## 5. Test Stand

**Figure 10:**
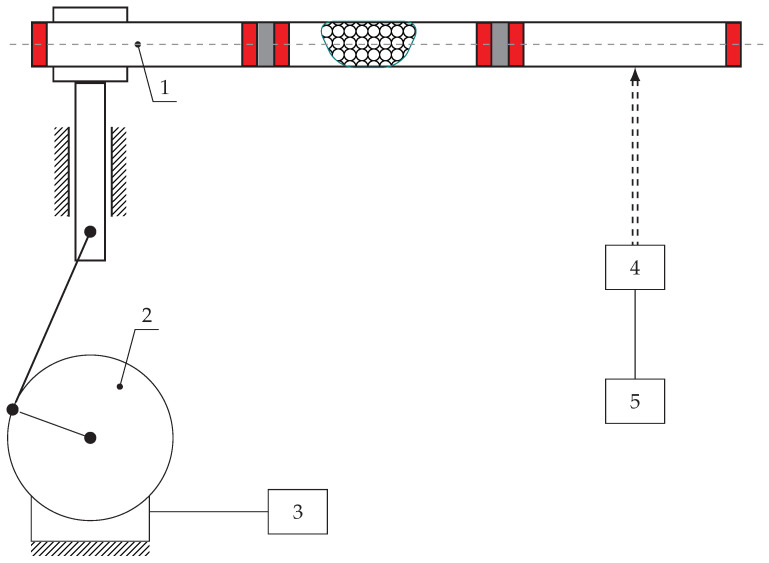
Scheme of the test stand (1—sponge particles beam; 2—motor; 3—inverter; 4—laser sensor; 5—data acquisition system).

In order to validate the mathematical model, the special research stand was designed. The main concept assumed a light and thin beam filled by special Sponge Particle Structure. In such a case SPS divides the beam into three separate sections by fixed elastic plates, balloons, and movable plates. Each section has a structure consisting of sponge sheets and uniformly distributed particles. The sponge parts are connected by glue, and the heavy steel grains are placed between them (Figure 10). Pumping the balloon allows one to move the sliding plates. As a result, sponges with particles in each section can change their volume and position which can be described as a mass redistribution. It was assumed that the masses of the SPS sections are much larger than the entire beam. It allows us to discretize the continuous system to three nodes. It corresponds to the model assumptions and simplifications adopted at the preliminary stage.

The designed test stand can be used for various types of the excitation. Vibration measurements with different distribution of masses along the beam were carried out. Test stand parameters are presented in Table 3 where $V_{max}$ means maximum volume of the balloon necessary for the redistribution of the whole grains mass to one node, and $P_{max}$ is the pressure of the balloon when the volume reaches its maximum value $V_{max}$.

**Figure 11:**
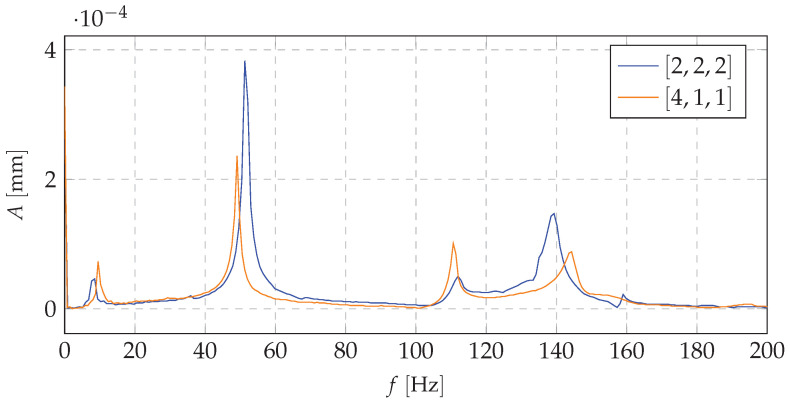
The amplitude spectrum of vibration accelerations (comparison of basic case and first mass distribution case.

**Figure 12:**
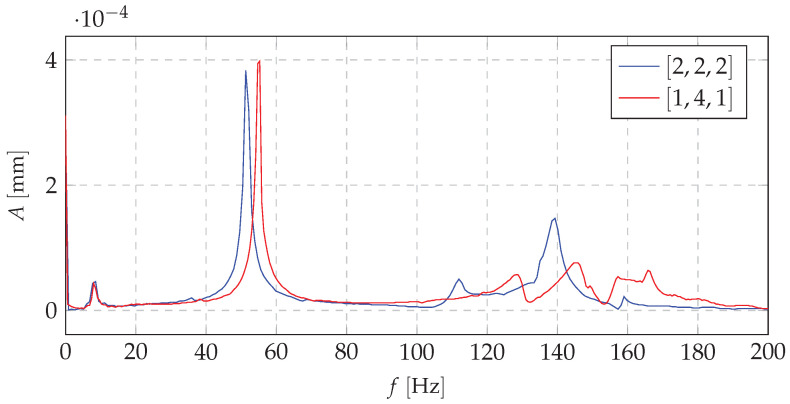
The amplitude spectrum of vibration accelerations (comparison of basic case and the second mass distribution case.

**Figure 13:**
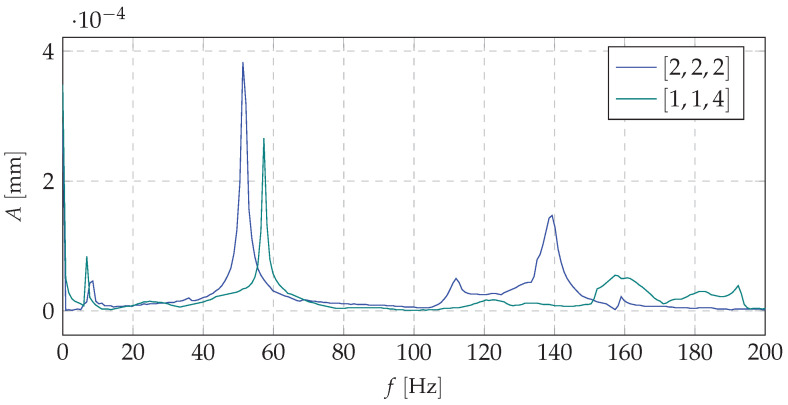
The amplitude spectrum of vibration accelerations (comparison of basic case and the third mass distribution case).

Figure 11, Figure 12 and Figure 13 show the amplitude spectra for the acceleration vibrations for various cases of the mass redistribution. Based on that, it is possible to compare the natural frequency of the beam for the initial case (uniform mass distribution) and for the mass being redistributed.

Analyzing the data presented in Figure 11, Figure 12 and Figure 13, it can be concluded that the proposed shaping method of the frequency structure causes the observable change in a dynamic behavior of the whole system. It can be stated that, for the each mass distribution case, there is a corresponding eigenfrequency which will change during the redistribution process. Increasing the mass density at a selected node allows one to change the natural vibration frequency up to 25% in comparison to the remaining configurations (distributions of the mass). Such changes in a frequency structure cause various frequency responses under the harmonic excitation. It means that, even if the system reaches its natural frequency, it is possible, thanks to the SPS, to change the mass distribution and, in consequence, limit the value of vibration amplitude. Moreover, theoretical and experimental analyses indicate that particular changes affect only selected eigenfrequencies. It is very important from the practical point of view because it allows us to control only a narrow band of the FRF. The remaining part of the spectrum will not be changed. It provides predictable dynamics of the system and makes the control process easier.

## 6. Summary

Slender elements, such as shafts, rods and beams, often work in a resonant range, in many engineering applications. Therefore, it is crucial to take into account this dangerous phenomenon in the design process. In this paper the authors proposed a novel method for adaptive–passive (semi-active) damping of vibration techniques, thanks to introducing an innovative structure based on granular materials. The paper also presents a discretized mathematical model, allowing for a quick determination of the natural frequency of a cantilever beam. This was applied to determine the value of frequency changes when 30% of the total mass of the structure was redistributed. Two theoretical methods of the mass relocation were taken into account: a step function and continuous change in the beam interior density. Both cases showed that there is a rational approach to the possible resonant range shifting. In addition, simulations of the dynamic mass redistribution allowed us to reveal a smooth transition from the initial to the expected frequency structure. Although the step change in vibrations is observed, the variations in a frequency structure do not appear and the system dynamic behavior is stable. Experimental tests carried out on the special test stand confirmed the correctness of numerical calculations. For the mass redistribution, not only SPS can be involved. Additionally, other unconventional structures, such as magnetorheological fluids or elastomers, can be useful [27,28]. The volume of the internal sections can also be changed mechanically (by screws), hydraulically or by the external magnetic field. Novel methods will be developed for a rapid mass change with the application of smart materials, such as Vacuum Packed Particles or magnetorheological fluids. Such an approach would allow for ’‘in real time” changes between different values of natural frequencies and vibration amplitudes.

## Figures and Tables

**Table 1:** Natural frequencies of beam for various discretization variants—uniform mass distribution.

No		3 Nodes	5 Nodes	7 Nodes	9 Nodes	10 Nodes
$f_{1}$	Hz	8.1	8.5	8.9	9.2	9.23
$f_{2}$	Hz	53.1	54.1	56.4	57.8	58.4
$f_{3}$	Hz	142.7	153.4	159.1	162.6	163.9

**Table 2:** A List of results of beam’s natural frequency for various discretization variants—the mass redistributed to the end of the beam.

No		3 Nodes	5 Nodes	7 Nodes	9 Nodes	10 Nodes
$f_{1}$	Hz	11	11.2	11.3	12.7	13.2
$f_{2}$	Hz	52.1	55	57.3	64.5	68.2
$f_{3}$	Hz	149.6	160.5	166.2	168.6	169.8

**Table 3:** Test stand parameters.

Component	Parameter	Value
Cantilever beam		Length: 400 mm
	Size	Inner diameter: 100 mm
		Outer diameter: 116 mm
	Material	Steel
Sponge	Density	$25\;kg/m^{3}$
	Material	Poliuretan
	Size	Diameter: 4 mm
Grains	Material	Steel
	Density	$7900\;kg/m^{3}$
	Size	$V_{max}=8.35\times 10^{-3}\;m^{3}$
Balloon	Pressure	$P_{max}\approx 100\;\mathrm{Pa}$
	Material	Rubber
